# Heterogeneity of Cancer‐Associated Fibroblasts and Precision Targeting Strategies for Cancer Therapy

**DOI:** 10.1111/1759-7714.70310

**Published:** 2026-05-28

**Authors:** Yijie Wang, Guangyao Zhou, Pengpeng Zhang, Zhenfa Zhang, Lianmin Zhang

**Affiliations:** ^1^ Department of Lung Cancer, Tianjin Lung Cancer Center, National Clinical Research Center for Cancer, Key Laboratory of Cancer Prevention and Therapy, Tianjin's Clinical Research Center for Cancer Tianjin Medical University Cancer Institute and Hospital Tianjin China

**Keywords:** cancer‐associated fibroblasts, extracellular matrix remodeling, immunotherapy, tumor escape, tumor microenvironment

## Abstract

Immune checkpoint blockade (ICB) has transformed cancer therapy, but its effect is often limited by immunosuppression and resistance mechanisms within the tumor microenvironment (TME). Cancer‐associated fibroblasts (CAFs) represent a major stromal component of the TME. They exhibit substantial heterogeneity, dynamic plasticity, diverse cellular origins, and context‐dependent activation states, which critically determine the safety and efficacy of CAF‐targeted therapies. CAFs contribute to tumor immune evasion by remodeling the extracellular matrix, altering tissue mechanics and establishing structural barriers. In addition, CAFs release cytokines, chemokines, and exosomes to impair T‐cell trafficking and function. They also interact with immune and myeloid cells, further reducing the efficacy of ICB. Therefore, CAF‐targeted therapies are shifting from the strategy of extensive clearance to a more elaborate framework, which focuses on specific CAF subpopulations and their regulatory pathways, and combines specific strategies for subpopulations, functional reprogramming, and methods based on advanced delivery systems and diagnostic tools. Integrating spatial omics, single‐cell technologies, and 3D culture systems can help us better understand the different states of CAFs and how they are distributed in space. This enables the identification of targetable CAF states, informs reprogramming strategies, supports rational combination design, and facilitates biomarker discovery. This review emphasizes the heterogeneity and plasticity of CAFs in tumor immunosuppression, and advocates a conceptual shift from static cellular identity to a dynamic state–niche–function framework.

## Graphical abstract

CAFs orchestrate tumor immunosuppression through spatially organized ECM remodeling, cytokine signaling, and metabolic reprogramming. Targeting CAFs requires a shift from marker‐based depletion to functional state and niche‐specific intervention.

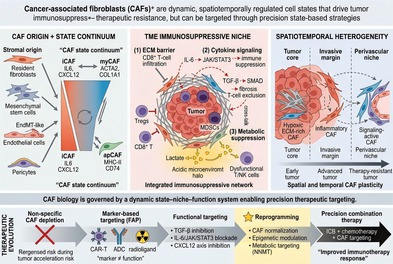

## 1 Introduction

Immune checkpoint inhibitors (ICIs) have transformed the treatment of many solid tumors. However, their overall clinical efficacy remains limited, largely due to the immunosuppressive tumor microenvironment (TME), which restricts the antitumor immune response and prevents immune effector cells from entering and functioning. In the TME, the extracellular matrix (ECM) not only acts as a physical barrier to immune cell infiltration but also serves as a dynamic signal platform to amplify immunosuppression. As the primary producers and remodelers of the ECM, CAFs promote desmoplasia, inhibit anti‐tumor immune activity, and lead to resistance to immunotherapy and other treatments [1, 2, 3]. Accordingly, CAF‐directed strategies are being explored to improve immunotherapy outcomes. However, the experimental results of early attempts to eliminate CAFs are not ideal and may even worsen the condition. These findings indicate that CAF biology is highly complex, rather than reflecting a lack of therapeutic potential per se [1, 2, 4]. CAFs comprise diverse and phenotypically plastic cell populations, and their functions depend on the environment, sometimes promoting tumors and sometimes inhibiting them. Their states dynamically change in response to the surrounding microenvironment, which makes indiscriminate depletion strategies ineffective [5, 6]. A deeper mechanistic understanding of CAF biology is therefore required to enable more precise therapeutic strategies.

## 2 The Cancer‐Associated Fibroblast Population in the Tumor Microenvironment: A Multidimensional Analysis of Heterogeneity, Classification, and Function

**FIGURE 1:**
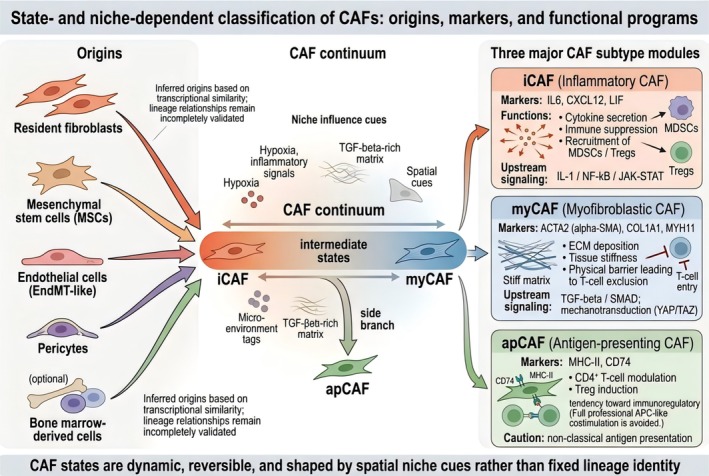
State‐ and niche‐dependent classification of CAFs: origins, markers, and functional programs.

CAFs are not the same kind of cells (Figure 1). In fact, there are multiple CAF subpopulations, and each subpopulation has its own unique function in the TME. Therefore, it is very important to study these differences to understand their complex functions. Recently, with the progress of single cell RNA sequencing (scRNA‐seq), scientists have found many subpopulations of CAF in different cancers. However, due to the different classification criteria used in various studies, these findings are difficult to verify repeatedly, which also limits their clinical application.

### 2.1 The Prevalence of Heterogeneity and Advances in Cancer‐Associated Fibroblasts Classification

CAFs are increasingly recognized as a highly heterogeneous stromal compartment whose diversity reflects not only lineage context but also dynamic state transitions within the TME. CAFs have been proposed to arise from resident tissue fibroblasts and, in some settings, from alternative stromal or lineage‐plastic populations under tumor‐induced pressure; however, in many human studies these proposed “origins” are inferred from transcriptional similarity and trajectory analysis rather than proven by definitive lineage tracing, and therefore should be interpreted cautiously [7, 8]. The traditional myCAF/iCAF binary framework has been useful for organizing early observations, but it is increasingly insufficient for precision oncology because it compresses continuous phenotypic variation, underestimates context dependence, and does not adequately capture the spatial organization of CAF states within tumors [7, 8].

Recent single‐cell and spatial‐omics studies have therefore shifted the field toward phenotype‐ and niche‐based classification. In a pan‐cancer single‐cell analysis of 226 samples across 10 solid tumor types, Luo et al. showed that major CAF populations follow a continuous activation trajectory and display substantial plasticity, while minor CAF fractions suggest possible alternative origins from endothelial or myeloid compartments and identify an endothelial‐to‐mesenchymal transition‐like CAF state associated with survival stratification [7]. Importantly, these findings are best interpreted as evidence for state continuity and shared transcriptional programs rather than as proof of a fixed lineage hierarchy, because trajectory inference and marker co‐expression alone cannot resolve true cell‐of‐origin relationships [7]. Complementing this view, Cords et al. integrated scRNA‐seq with highly multiplexed imaging mass cytometry and proposed nine CAF phenotypes plus pericytes, validating the classification across four additional cancer types and confirming the spatial distribution of these phenotypes at the protein level [9]. This study is particularly valuable because it links transcriptomic states to spatial context; however, its framework should be regarded as a pragmatic working model rather than a universal ontology, since the number and boundaries of CAF subpopulations depend on clustering resolution, marker availability, sample composition, and the risk of overlap with pericyte or myeloid signatures [8, 9].

More recently, large‐scale single‐cell spatial multi‐omics studies have further supported the existence of conserved spatial CAF subpopulations across cancer types and have shown that CAF abundance and neighborhood composition are associated with immune phenotypes and patient survival, reinforcing the idea that spatially resolved CAF states may be more clinically informative than marker‐based labels alone [10]. Nevertheless, a comprehensive and functionally validated CAF classification system remains unresolved; the central challenge is not simply to enumerate more subpopulations, but to define reproducible, causally linked, and therapeutically actionable CAF programs that can be translated across platforms and tumor contexts [8, 10, 11].

### 2.2 Functional Diversity Across and Within Tumor Types

CAFs represent a heterogeneous stromal cell population in the TME, and are better conceptualized as a dynamic cellular state, rather than a fixed cell [12, 13]. They come from different precursor cells, such as fibroblasts and other stromal cells, and their behavior will be influenced by tumor and surrounding environmental signals. Therefore, the characteristics and functions of CAFs are highly heterogeneous; consequently, not all CAFs promote tumor progression. A key feature of CAFs is that their functions are highly context‐dependent. According to the specific tissue environment, CAFs may play completely different or even opposite roles in different cancers. For example, in intrahepatic cholangiocarcinoma, CAFs mainly promote tumor development through paracrine signaling and ECM remodeling [14]. On the contrary, in colorectal cancer, a specific CAF subpopulation can prevent tumor progression by inhibiting the epithelial–mesenchymal transition (EMT), which indicates that CAFs do not always promote tumor progression [15]. Similarly, in pancreatic cancer, some CAF subpopulations are tumor promoters, while others play a role in tumor inhibition, which emphasizes that their effects are highly dependent on local environmental conditions [13, 16].

Within individual tumors, CAF diversity is further shaped by spatial organization and local signaling environments. Different CAF subpopulations, such as inflammatory CAFs (iCAFs) and myofibroblastic CAFs (myCAFs), have their own special tasks: iCAFs are mainly responsible for cytokine communication and immune response, while myCAFs participate in building extracellular matrix and tissue structure. However, these functional identities are not static, and they will change according to the signals coming from around them, which shows that the functions of CAF are flexible and not predetermined by their cell origins [15, 16].

Importantly, CAFs can do more than just promote tumor progression. Although there are many activities of CAFs that can promote tumor progression, help cancer cells avoid the immune system and make treatment difficult, there are also some other types of CAF that can inhibit tumor development or maintain the normal structure of the host tissues. For example, collagen‐producing CAFs can physically limit the spread of tumors; in addition, Meflin‐positive CAFs have been associated with better prognosis in pancreatic cancer [17, 18].

CAFs exhibit highly dynamic and context‐dependent functional states across tumor types and within individual tumors, reflecting their intrinsic biological complexity. Some will promote tumor progression, while others can inhibit it. This natural diversity is the fundamental reason for the biological complexity of CAF, and it also has a great influence on our study of them and ways to treat them [12, 19].

## 3 The Central Role of Cancer‐Associated Fibroblasts in Immunosuppression and Treatment Resistance

**FIGURE 2:**
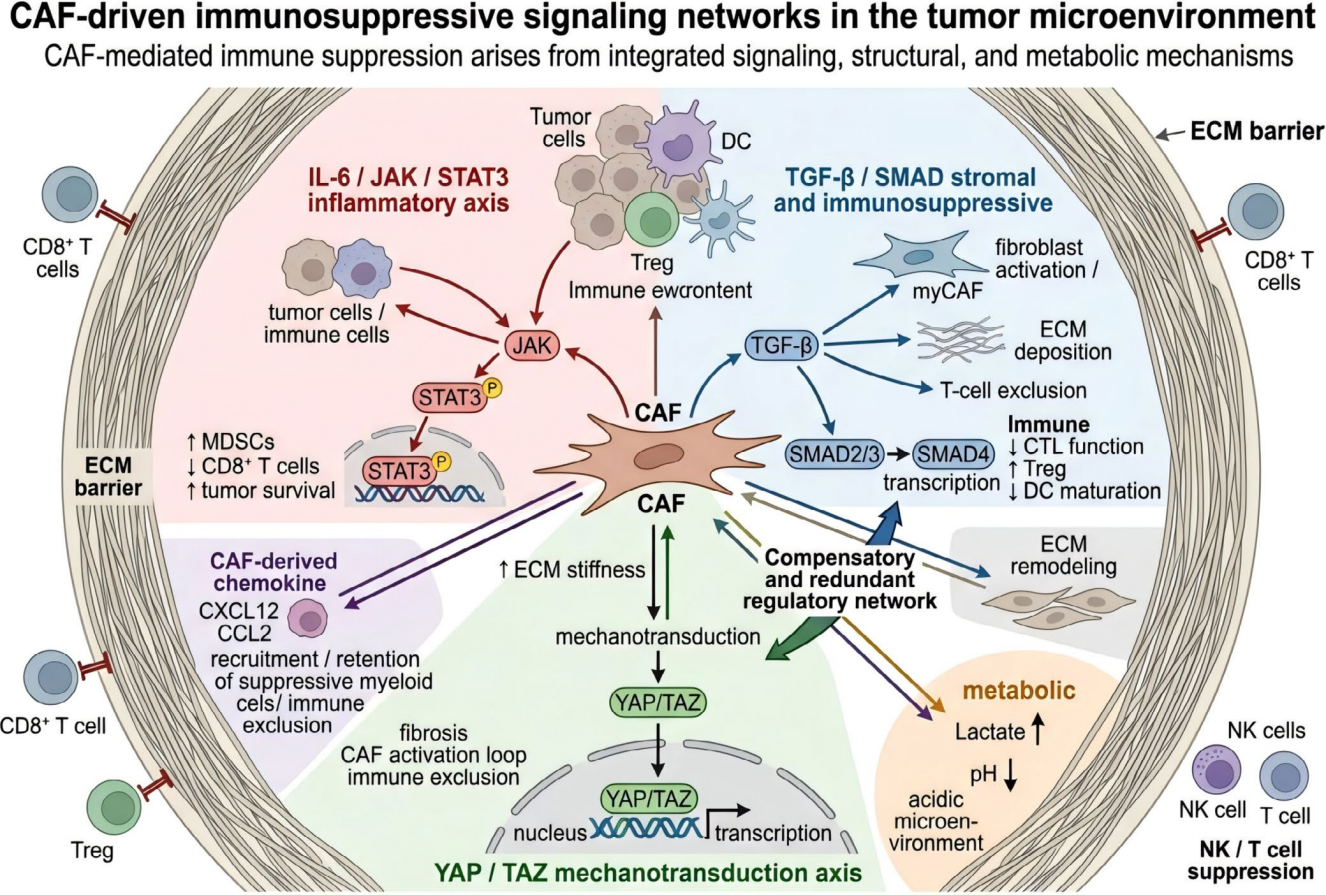
CAF‐driven immunosuppressive signaling networks in the tumor microenvironment.

CAFs play critical roles in mediating tumor immune evasion and therapy resistance [3, 20]. They establish an immunosuppressive TME through multiple mechanisms (Figure 2), including forming physical barriers, directly suppressing immune cell function, and remodeling metabolic conditions within the TME [21, 22].

### 3.1 Extracellular Matrix Remodeling and Immune Exclusion

CAFs synthesize and remodel ECM components, forming dense fibrous structures in the tumor [23, 24]. These structural changes not only change the mechanical properties of tissues, but also affect the behavior of immune cells through the exchange of physical, chemical, and mechanical signals. ECM is not a static barrier; it is actually an active signal center, regulating immune cell localization and function [25, 26].

First, CAF‐driven ECM deposition physically constrains immune cell infiltration. In non‐small cell lung cancer, distinct CAF subpopulations—MYH11^+^αSMA^+^ and FAP^+^αSMA^+^—occupy specific spatial regions and secrete ECM components such as type IV and type XI/XII collagens, forming dense stromal barriers that restrict CD8^+^ T‐cell penetration. Similarly, in squamous cell carcinoma models, myCAF subpopulations enriched for ECM genes (e.g., COL11A1) are associated with exclusion of T cells to the tumor periphery. These findings support the role of ECM architecture in defining immune‐excluded tumor phenotypes [25, 27].

However, the physical barrier alone cannot fully explain immune exclusion. Extracellular matrix will also control the distribution and activity of those signal proteins. ECM components such as proteoglycans and glycosaminoglycans can bind and immobilize chemokines (such as CXCL12), thus forming a spatial concentration difference. These chemokine gradients will trap T cells in the matrix area and prevent them from running into the tumor mass. This process of immobilizing chemokines not only strengthens the spatial separation of immune cells, but also helps to create the “immune‐excluded” microenvironment [25, 27].

Moreover, the key to the movement of T cells in tumor matrix depends on whether they can combine with the components of extracellular matrix through integrin. Excessive matrix stiffness and crosslinking reduce ligand availability for integrin engagement, thereby impairing T‐cell adhesion and directional migration. As a result, although there are T cells in the tumor environment, they exhibit impaired motility and can't effectively contact tumor cells [25, 28].

The remodeling of extracellular matrix will greatly change the stiffness and mechanotransduction signaling of tissue. More collagen deposition and cross‐linking will make the tissue harder, thus activating mechanotransduction pathways (such as YAP/TAZ‐related transcription processes) in stromal cells and tumor cells. These pathways will make CAFs more active, stimulate them to produce more extracellular matrix, and help maintain the immunosuppression signal network. It is particularly noteworthy that the stiffness of the matrix will also affect the behavior of immune cells, further weakening their killing function [25, 27].

Taken together, this evidence reveals that the extracellular matrix remodeling driven by CAFs will promote immune rejection through structural barriers, gradient manipulation of chemokines, and stiffness‐triggered mechanical conduction. These interrelated processes occur in a specific microenvironment, especially at the tumor‐matrix interface. Importantly, although the accumulation of extracellular matrix may promote immune evasion, it may also physically hinder tumor growth in some cases. Therefore, the treatment strategy should focus on regulating rather than completely eliminating extracellular matrix, so as to balance its dual effects on tumor progress and control [25].

### 3.2 Paracrine Signaling and Suppression of Immune Cell Function

CAFs establish a complex immunosuppressive network in the TME, which release a broad range of immunosuppressive cytokines and chemokines that can modulate immune cell function [3, 21, 22]. Among these factors, transforming growth factor‐beta (TGF‐β) is a particularly powerful immunosuppressant. TGF‐β from CAFs will weaken the function and killing ability of cytotoxic T lymphocytes (CTLs), promote the formation of regulatory T cells, and inhibit the maturation of dendritic cells, thus greatly weakening our adaptive immune response [29]. In addition, experimental studies have proved that TGF‐β produced by CAF will directly impair the function of tumor‐infiltrating lymphocytes (TILs) [21].

Besides TGF‐β, interleukin‐6 (IL‐6) secreted by CAFs is another key immunosuppressive factor. IL‐6 promotes tumor progression and survival by activating the JAK/STAT3 pathway, while also reprogramming immune cells toward inhibitory phenotypes [22, 30]. For example, it increases the number and activity of myeloid‐derived suppressor cells (MDSCs) and reduces CD8^+^ T‐cell function. Furthermore, CAFs release chemokines such as CXCL12 and CCL2, which recruit Tregs, MDSCs, and tumor‐associated macrophages (TAMs) to the tumor site [21, 31]. These chemokines form a positive feedback loop that amplifies immunosuppressive responses and further consolidates the immune inertness of the TME.

Certain types of CAFs, such as antigen‐presenting CAFs (apCAFs), will affect the differentiation of CD4^+^ T cells in various cancers. These cells bind to T cell receptors through MHC molecules, which often promote the formation of immunosuppressive regulatory T cells (Tregs), which can help tumors avoid the discovery of the immune system [32]. In addition, CAFs will also release some effector molecules such as IL‐33 and GM‐CSF, which are crucial for controlling the polarization of TAMs, the accumulation of MDSCs, and the impairment of NK cell function [3, 22].

In brief, CAFs have established a complex immunosuppressive system. This system can affect different immune cells through various signaling mechanisms [3, 21]. It will weaken the immune system's ability to fight tumors, and it will also make treatments like immune checkpoint blockade less effective [29]. Therefore, it is very important to interrupt these immunosuppressive processes led by CAFs. Usually, these signal pathways will form a powerful immunosuppressive network, and CAFs play a core role in this network.

### 3.3 Metabolic Reprogramming and Immune Microenvironment Remodeling

CAFs in the TME undergo profound metabolic reprogramming, and they mainly change from oxidative phosphorylation to glycolysis. This kind of metabolic reprogramming that happens to CAFs will lead to a large number of lactate production and accumulation [33, 34]. Although this process can provide energy and materials for nearby cancer cells through “Metabolic symbiosis”, a more important result is the formation of an immunosuppressive microenvironment. Studies have shown that lactate can reduce the pH value of local tissues and disturb the important glycoenzymes, which will inhibit the proliferation and activity of CD8^+^ T‐cells and NK cells [35]. In addition, lactate also functions as a signaling metabolite, which can promote tumor‐related macrophages to adopt an immunosuppressive M2‐like phenotype, which further weakens the antitumor immunity [36].

In a cancer with severe fibrosis, such as pancreatic ductal adenocarcinoma (PDAC), CAFs will undergo great metabolic changes, mainly due to the hypoxic TME. The process of their conversion to glycolysis is closely related to the activity of hypoxia‐inducible factors and the inflammatory response of tumors [34]. CAFs can also affect the behavior of immune cells through metabolic processes. An obvious example is the increased expression of nicotinamide *N*‐methyltransferase (NNMT) in CAFs, which will interfere with the availability of methyl and produce *N*‐methylnicotinamide, thus inhibiting the activity of CD8^+^ T cells and promoting immune evasion [37]. In summary, these observations underscore that CAF‐driven metabolic adaptation is a key driver of tumor metabolic network reorganization and promotes the formation of an immunosuppressive TME. These observations suggest that the metabolic reprogramming of CAFs promotes immunosuppression through many interrelated ways.

## 4 New Therapeutic Strategies Targeting Cancer‐Associated Fibroblasts

**FIGURE 3:**
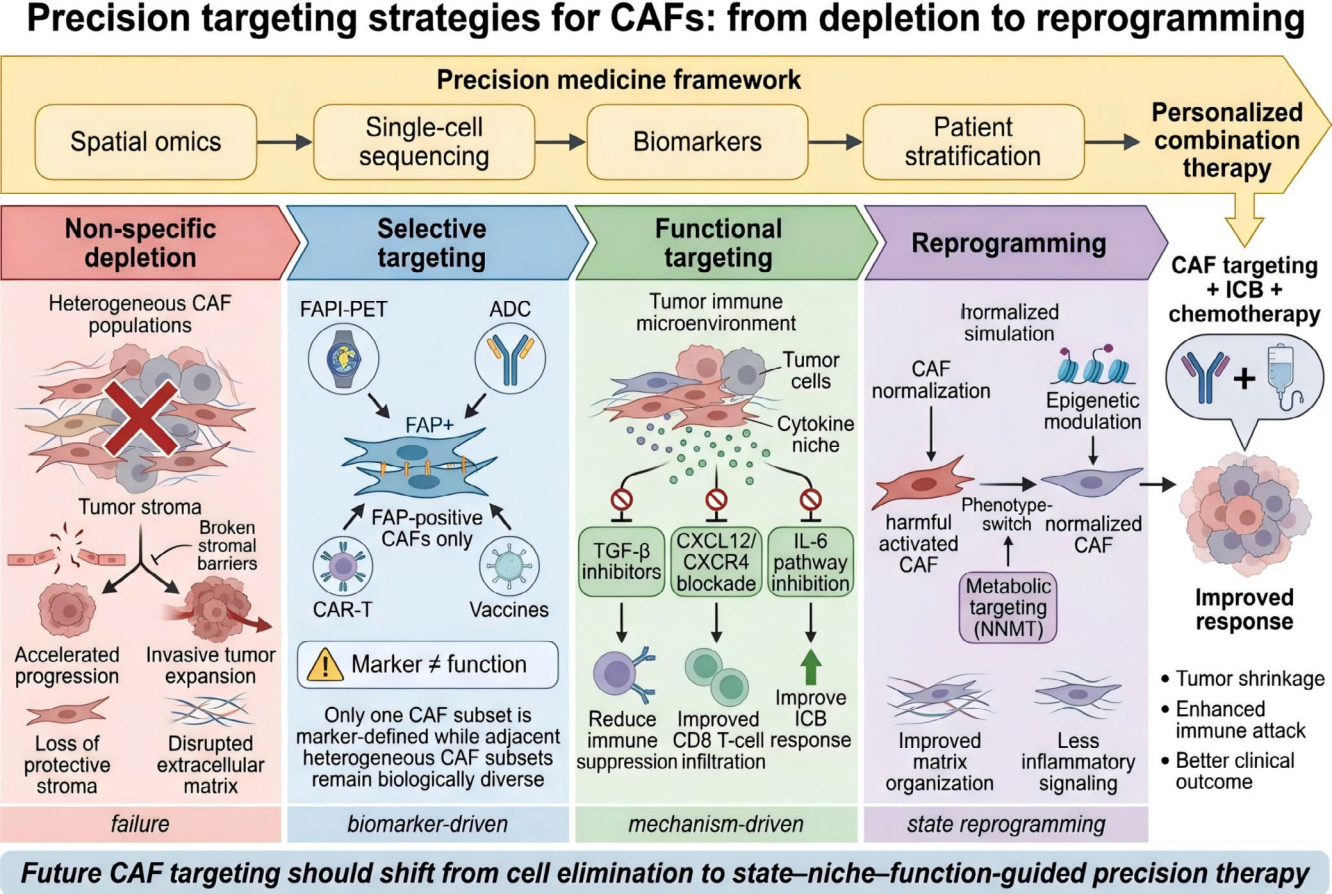
Precision targeting strategies for CAFs: From depletion to reprogramming.

Previous attempts to eliminate or inhibit CAFs indiscriminately have failed in animal experiments and clinical research (Figure 3). Some therapies have shown limited efficacy and, in certain contexts, may even accelerate tumor progression and weaken the immune system's immunity against tumors [38, 39]. Such results underscore the notion that CAFs do not all promote tumor progression; in fact, their role in the TME is very complicated, depending on the specific situation. Therefore, the focus of scientists' work has changed from thinking about completely eliminating CAFs to more elaborate methods, such as distinguishing tumor‐promoting from tumor‐restraining CAF subpopulations, and then focusing on those harmful cells or key molecular pathways [20].

### 4.1 Precision Clearance via Molecular Markers for Tumor‐Promoting CAF Subpopulations

Early therapeutic strategies aimed at broadly depleting stromal components or targeting CAFs indiscriminately. However, the clinical effect of this method is limited because CAFs are highly heterogeneous. They sometimes promote tumor progression and sometimes inhibit tumor development, depending on the situation. Moreover, this indiscriminate removal of matrix will destroy the tissue balance, and it does not take into account the functional differences of different CAF subpopulations [40, 41].

In this case, CAFs expressing fibroblast activation protein (FAP) have become an important way of precise interstitial therapy. FAP is highly expressed in CAFs of various solid tumors, but rarely expressed in most normal adult tissues, which makes it an ideal target for selective intervention [41, 42]. Therefore, FAP targeting strategy has been extended from the traditional antibody method to include radioligands, immunotoxins, antibody‐drug conjugates (ADCs), vaccines, CAR‐T cells and photoimmunotherapy. Pre‐clinical and early clinical data show that FAP targeted drugs can selectively accumulate in tumor stroma, which may enhance the effect of immune checkpoint inhibition or other cancer therapies [43, 44, 45].

Several molecular markers have emerged as potential candidates for such precision clearance. For example, CAFs characterized by CD10 and GPR77 will sustain cancer stemness and chemoresistance; targeting GPR77 suppresses tumor growth and restores chemosensitivity. Similarly, senescent CAF subpopulations will inhibit the immune system and cause inflammation. If we selectively remove them, such as using BCL‐2 inhibitors, we can enhance the combat effectiveness of CD8+ T cells and make immunotherapy more effective. Generally speaking, these findings tell us that the treatment for CAFs will not simply eliminate them all, but accurately eliminate those tumor‐promoting CAF subpopulations [40, 46].

So, the therapeutic goal is increasingly shifting from non‐specific CAF depletion toward the precise elimination of tumor‐promoting CAF subpopulations defined by molecular markers. However, even precise elimination represented by the FAP^+^ CAF subpopulations may lead to mixed or even contradictory results. The reason is that the FAP+ compartments do not promote tumors uniformly, and some spatially defined CAF subpopulations can inhibit tumor progression or support tissue integrity. A critical limitation of the field is that marker identity is not equivalent to functional identity. FAP, CD10, GPR77, and related markers can enrich for pathogenic CAF states, but they do not define them universally across tumor types or stages. Spatial studies in NSCLC and immunotherapy cohorts further show that fibroblast location and neighborhood structure correlate with immune exclusion and outcome, underscoring that CAF biology is context‐dependent rather than marker‐determined [47, 48].

A key unresolved challenge is that even with refined molecular markers, we still cannot eliminate cancer‐promoting CAF components accurately. To achieve precise targeting, it is necessary to understand the transcriptional regulatory network upstream and downstream.

### 4.2 Transcriptional Regulatory Networks and Precision Targeting of Cancer‐Associated Fibroblasts

Due to the failure of precision‐targeting approaches, an alternative approach to treating CAFs has been proposed, which views it as a highly plastic matrix state rather than a fixed cell type. Recent single‐cell and spatial studies show that CAF heterogeneity is not random: it is organized by conserved niches, local cell–cell interactions, and disease‐specific transcriptional programs. In pancreatic cancer, the invasive front is enriched for TNFα/NF‐κB, hypoxia, glycolysis, and EMT‐associated stress programs in both cancer and CAF compartments, while pan‐cancer spatial profiling has identified conserved CAF subpopulations and spatial neighborhoods across tumor types [49, 50]. Similarly, single‐cell spatial transcriptomics indicates that immunotherapy response is associated more with the balance of immune‐supportive versus immunosuppressive fibroblast states than with CAF abundance per se [48]. Spatial omics frameworks further support the idea that persistent neighborhood patterns can outperform marker‐only classification in predicting tissue behavior and treatment response [51].

At the regulatory level, four recurrent regulatory axes consistently structure CAF state transitions: IL‐6/JAK/STAT3, TGF‐β/SMAD, NF‐κB, and mechanotransduction‐associated transcriptional programs. IL‐6/JAK/STAT3 sustains inflammatory CAF states and promotes chemokine‐rich, therapy‐resistant secretory phenotypes; TGF‐β/SMAD biases CAFs toward myofibroblastic, matrix‐producing programs that reinforce stromal stiffness and T‐cell exclusion; and NF‐κB integrates inflammatory inputs from tumor cells and myeloid cells to amplify cytokine output and adaptive resistance. Importantly, these pathways should not be treated as isolated targets. They form a compensatory network, which helps explain why single‐node inhibition has repeatedly underperformed in the clinic.

This web‐based view has changed the way we develop treatments. The first strategy is to reprogram or restore the cells to normal, and the focus is on inhibiting pathogenic transcriptional states, rather than destroying all fibroblasts. In fact, this includes inhibiting TGF‐β or CXCL12/CXCR4 signals, blocking inflammatory pathways, and combining matrix regulation with immune checkpoint blocking. These interventions have achieved good results and proved that this treatment method is feasible.

Therefore, the therapeutic paradigm now is no longer to completely eliminate CAFs, but to find ways to change their behavior. Under this new idea, CAFs are more like a complex regulatory network, which needs to be carefully adjusted rather than simply eliminated. However, many models used in laboratory research are too simple, but there are actually various CAF subpopulations in real human tumors, and their functions may be completely opposite, and many signal pathways have backup mechanisms. This suggests that we have to choose the treatment target according to the specific solid tumor, and we may have to block several signal pathways at the same time to achieve the desired effect [52, 53].

### 4.3 Spatiotemporal Heterogeneity as a Unifying Framework for Cancer‐Associated Fibroblasts Biology

Increasing evidence from spatial multi‐omics and single‐cell studies indicates that the diversity of CAFs is not only due to specific protein markers but also related to their location and time of appearance. Although labels such as FAP, ACTA2, and COL1A1 can help us recognize the status of some CAFs, they do not completely determine what CAFs do. The characteristics of CAF are actually shaped by the local environment around them, such as organizational structure, nearby cells, and signal molecular networks. In addition, we have found similar subpopulations of CAF in different cancers and with different spatial omics techniques, which shows that “spatial identity” is a key factor to determine the function of CAF [10, 54].

CAFs are arranged along a continuous space instead of staying in separate compartments. From the tumor core to the edge of its invasion, it forms a continuous spatial gradient: at one end, there are many ECM, little oxygen, and immune cells are excluded. At the other end lies the tumor–stroma interface, where CAFs, tumor cells, immune cells, and blood vessels mix and influence each other. The study of spatial transcriptome of solid tumor found that this place at the junction of tumor and matrix formed a unique ecological state, where there was a specialized matrix microenvironment and the immune microenvironment became different. This shows that the invasion front of tumor is not only a transitional zone, but a functional niche [55, 56, 57].

At the edge of tumor invasion, CAFs can coordinate the EMT, immunosuppression, and matrix remodeling, which makes this region a key position for tumor development and treatment resistance. Through the analysis of spatially resolved protein transcriptome, we also found that CAF‐driven ECM reconstruction and cancer‐matrix collaboration are mainly concentrated at the tumor‐matrix junction, where signal pathways may link the processes of matrix deposition, invasion, and immune rejection [58, 59].

In this special area around blood vessels, CAFs are like a part of a large vascular‐immune‐interstitial system. They can not only make extracellular matrix, but also regulate the behavior of endothelial cells and the transport of immune cells, and can flexibly switch between the two functions of promoting tumor and inhibiting tumor according to the signals of the surrounding environment. A space omics review on perivascular tumor ecosystem emphasizes that the characteristics of this region are determined by the cooperation among vascular cells, fibroblasts, and immune infiltration, not just the proximity to blood vessels [54, 57].

In addition to the spatial tissue, the state of CAF will also change dynamically with the development of tumor and the pressure of treatment. This temporal plasticity explains why the effect of CAF clearance or single pathway inhibition strategy is unstable. From the methodological point of view, it also shows that spatial transcriptomics alone is insufficient, because transcription localization is not necessarily related to protein's functional activities, so it is necessary to combine multi‐genomics methods. In fact, many studies have found that the amount of mRNA in stroma is inconsistent with the expression of protein, which further proves that the function can be accurately inferred only by combining spatial transcriptomics, spatial protein omics or multiple imaging [58, 60, 61].

## 5 Summary and Outlook

CAFs are increasingly recognized as highly heterogeneous and plastic. However, the popular method of classifying them by “subpopulations” is actually insufficient. More and more single cell and spatial data show that the functional states of CAFs are not completely different, but more like a continuously changing spectrum, which will change dynamically with the different surrounding environment. Therefore, expanding CAF classification alone is insufficient for clinical translation without functional and spatial validation, which actually points to a more fundamental problem: in this field, identities defined by markers are often confused with their actual functional behaviors [52, 62].

This fundamental difference may be why the treatment for CAFs always fails. Markers like FAP or ACTA2 can help us identify specific cell types, but they can't tell us exactly what these cells do. Moreover, if we only block one signal path, other paths will make up. The immunosuppression caused by CAF is actually caused by a spatial order regulation system. In this system, the remodeling of ECM, the signal of cytokines and the metabolic reprogramming of cells are closely intertwined and change together, especially in such a specialized microenvironment as the junction of tumor and matrix [63, 64, 65].

These limitations make the classification method shift from cell‐centered to system‐level framework. After the axis of “state‐microenvironment‐function” is extended to the model of “regulatory network‐microenvironment coupling,” the cancer‐related fibroblast state can be regarded as a dynamic systems‐level attractor governed by interconnected regulatory circuits. From this point of view, the effective treatment method should not try to eliminate fibroblast subpopulations, but should adjust the network stability by combining spatial information strategies, so as to solve the pathological state.

Future progress will no longer depend on identifying additional CAF subpopulations, but on finding the core laws that determine the behavior of matrix systems. Combining spatial multi‐omics with functional verification is the key to connecting descriptive observation with causal view. Finally, we should regard CAFs as a dynamically reprogrammable element in the tumor ecosystem, so as to promote the treatment strategy based on biomarker guidance and specific situations.

## Author Contributions

**Yijie Wang:** conceptualization, methodology, investigation, formal analysis, visualization, writing – review and editing. **Guangyao Zhou:** writing – review and editing, investigation, data curation, software. **Pengpeng Zhang:** investigation, data curation, funding acquisition, writing – review and editing. **Zhenfa Zhang:** writing – review and editing, supervision, methodology, conceptualization. **Lianmin Zhang:** writing – review and editing, supervision, project administration, resources, funding acquisition.

## Funding

The authors have nothing to report.

## Ethics Statement

This article is a literature review and does not involve new experimental research involving humans or animals, and therefore does not require ethical committee approval. All studies cited in this article that contain data from human subjects have declared in their original research that appropriate ethical committee review and approval were obtained, and that patient informed consent was secured. The authors solemnly declare that they are responsible for the originality and accuracy of the citations in this manuscript, and that there is no academic misconduct of any kind.

## Conflicts of Interest

The authors declare no conflicts of interest.

## Data Availability

Data sharing not applicable to this article as no datasets were generated or analysed during the current study.
